# Expression of an Androgenic Gland-Specific Insulin-Like Peptide during the Course of Prawn Sexual and Morphotypic Differentiation

**DOI:** 10.5402/2011/476283

**Published:** 2011-04-11

**Authors:** Tomer Ventura, Rivka Manor, Eliahu D. Aflalo, Simy Weil, Isam Khalaila, Ohad Rosen, Amir Sagi

**Affiliations:** ^1^Department of Life Sciences, Ben-Gurion University of the Negev, P.O. Box 653, Beer Sheva 84105, Israel; ^2^National Institute for Biotechnology in the Negev, Ben-Gurion University of the Negev, P.O. Box 653, Beer Sheva 84105, Israel; ^3^Department of Biotechnology Engineering, Ben-Gurion University of the Negev, P.O. Box 653, Beer Sheva 84105, Israel

## Abstract

The crustacean male-specific androgenic gland (AG) regulates sexual differentiation. In the prawn *Macrobrachium rosenbergii*, silencing an AG-specific insulin-like encoding transcript (*Mr-IAG*) inhibited the development of male sexual characters, suggesting that Mr-IAG is a key androgenic hormone. We used recombinant pro-Mr-IAG peptide to generate antibodies that recognized the peptide in AG cells and extracts, as verified by mass spectrometry. We revealed the temporal expression pattern of *Mr-IAG* and studied its relevance to the timetable of sex differentiation processes in juveniles and after puberty. *Mr-IAG* was expressed from as early as 20 days after metamorphosis, prior to the appearance of external male sexual characters. *Mr-IAG* expression was lower in the less reproductively active orange-clawed males than in both the dominant blue-clawed males and the actively sneak mating small males. These results suggest a role for Mr-IAG both in the timing of male sexual differentiation and in regulating reproductive strategies.

## 1. Introduction

Sexual differentiation in the animal kingdom can be defined as a series of events whereby the sexually indeterminate embryo progressively acquires male or female characteristics in the gonads, genital tract, and external genitalia. Sexual determination and differentiation are highly diverse processes that have evolved independently numerous times [1]. Normal sexual development in gonochoristic species consists of several sequential stages. Genetic sex, as determined by the chromosome constitution or other factors, or a combination of the two, drives the primitive gonad to differentiate into a testis or an ovary.

In mammals, internal and external genitalia will subsequently follow the male pathway in the presence of specific testicular hormones or the female pathway in their absence [2]. Anti-Mullerian hormone (AMH) and testosterone are the two key hormones produced by the testes in optimal concentrations during a critical time frame in early gestation to ensure male development. These two hormones are produced in parallel to the developmental expression of their cognate receptors in target tissues [3]. In addition to these two factors, an array of peptidic and steroidal sex hormones governs the process of sexual differentiation in all vertebrates, but with differences between and within phyla.

In reptiles, for example, where temperature-dependent sexual determination predominates, and likewise in birds, sexual differentiation is primarily based on levels of circulating estrogen [4–6]. This mechanism differs from that in fish, in which there is a variety of differentiation processes, even in gonadogenesis itself. While in some species differentiation of the gonad into either a testis or an ovary is triggered by steroids, in other species, an undifferentiated ovary-like gonad develops, which later degenerates in half of the population [7–9].

In insects, the regulation of the sexual differentiation mechanism is subject to debate. The well-studied sexual determination and differentiation mechanisms in *Drosophila* favor the belief that there are no insect sex hormones, as the above mechanisms are thought to rely solely on autonomous cellular differentiation. There are, however, reports of the steroidogenic effect of gonadotropins in several other insect species [10]. In the closely related crustaceans, which are thought to have speciated early amongst the wide group of arthropods [11, 12], there is a unique androgenic gland (AG) that regulates both male sex differentiation and male reproductive physiology, and since, unlike vertebrates, the endocrine and gametogenic functions are clearly separated into distinct organs, the AG and the testis, respectively [13].

In research spanning several decades, the functioning of the AG has been investigated in a number of crustacean species by following the morphological and physiological effects on primary and secondary sex characteristics of AG removal or transplantation into females [14, 15]. In the giant freshwater prawn, *Macrobrachium rosenbergii*, for example, a degree of masculinization was recorded in AG-implanted females [16]. In the same species, fully functional sex reversal from males to neofemales [17] and from females to neomales [18] was achieved by bilateral AG ablation or transplantation, respectively. 

It has been hypothesized that the AG of decapod crustaceans secretes the hormone(s) responsible for male sexual differentiation, with accumulating ultrastructural and histological evidence suggesting that the hormone(s) is(are) proteinaceous in nature [19, 20]. The first reported AG hormone in Crustacea, which was found in isopods, was assigned to the insulin family of hormones, since it possesses B and A chains with a skeleton of conserved cysteine residues, connected by a C peptide that is present in the prohormone but undergoes cleavage to give rise to the mature hormone [21, 22]. In keeping with these findings, the establishment of an AG cDNA subtractive library in the decapod crayfish *Cherax quadricarinatus *revealed an insulin-like gene [23]. This result, in turn, paved the way for the discovery of *M. rosenbergii* insulin-like androgenic gland factor, designated *Mr-IAG*. Silencing of *Mr-IAG* in males impeded spermatogenesis and male secondary sexual characteristics [24], a finding that makes *Mr-IAG* a leading candidate for being an AG hormone.

In previous studies by our group, fully functional sex reversal of *M. rosenbergii* males into neofemales by AG ablation was achieved as late as 60 days after metamorphosis (60 days after larvae; PL_60_), but better success rates were achieved when the ablation was performed earlier at PL_20-30_ [25]. These findings suggest that in* M. rosenbergii,* not only does sex differentiation reach a “point of no return” by this time (PL_60_), but particularly that the AG plays a crucial role governing male sexual differentiation. Aflalo et al. [25] identified juvenile males according to the appearance of the appendix masculina, a male secondary sexual characteristic projecting from the second pleopod of *M. rosenbergii* males. This feature develops in males between 20 and 120 days after metamorphosis, depending on the size and weight of the prawn, parameters that are highly dependent on environmental traits such as stocking density (Aflalo, personal communication). One can speculate that the endocrine regulation of sexual differentiation precedes the phenotypic appearance of this secondary external feature.

In the animal kingdom, alternative mating strategies are widespread and arise in species where mating opportunities are skewed such that a small proportion of males in a population acquire mates, and there is substantial intrasexual variation in sexually selected traits, driving the evolving of subordinate males with distinct morphology or behavior to enhance their fitness [26]. In vertebrates, several studies have demonstrated that dominance is correlated with elevated levels of testosterone in the plasma, and the variation among morphs arises through feedback loops in the hypothalamic-pituitary-gonadal axis [27, 28]. It has been suggested that in crustaceans, methyl farnesoate (MF), an unepoxidated form of juvenile hormone III that controls reproduction in insects, plays the same role as testosterone in vertebrates. In the spider crab *Libinia emarginata*, for example, MF hemolymph levels were positively correlated with reproductive activity in males. In morphotypes exhibiting high reproductive indices (including both the primary reproductive large, long-clawed abraded males and the smaller abraded, short-clawed males that use tactics such as sneak-mating and female mimicry), MF hemolymph levels were twice as high as those of the reproductively inactive morphotypes, which include nonabraded males of all sizes [29]. In *M. rosenbergii*, however, there was no correlation between MF hemolymph levels in mature males and morphotypic reproductive activity [30]. *M. rosenbergii* males progress through a succession of male morphotypes, from subordinate small males through orange-clawed males to the dominant blue-clawed males. Mature *M. rosenbergii* males can be categorized into these different morphotypes on the basis of a number of external characters [31]. The clearly distinct morphotypes differ in their reproduction-related behavior and physiology. The small males practice a form of active sneak mating, consistent with their small size and high mobility, the orange-clawed males demonstrate a reduced rate of reproductive activity in the presence of dominant males, and the dominant blue-clawed males, which are usually the largest males, actively court and protect receptive females prior to mating [32]. As may be expected from their behavior, the subordinate small males and the dominant blue-clawed males were found to have a higher gonadosomatic index (GSI) than that of the orange-clawed males, with the GSI values of the subordinate small males being similar to those of the dominant blue-clawed males [33]. 

Since the AG has a marked effect on the primary and secondary male characteristics, it has been suggested that this organ has dual functions—both in male sex differentiation and in the maintenance of male morphological and anatomical features—a premise supported by the study by Sagi et al. [34] that demonstrated that the AG is necessary for morphotypic progression in *M. rosenbergii*. It is, therefore, important to identify an AG-borne factor whose levels vary according to the morphotypic progression. It has previously been suggested that certain polypeptides in the AG of *M. rosenbergii* were AG hormones on the basis of their higher levels in dominant blue-clawed males than the orange-clawed males and of their size similarity with isopod AG hormones (*∼*16, *∼*18 kDa) [35]. However, their sequences were not revealed, and therefore, they could not be related to *Mr-IAG*.

Since *Mr-IAG* is a leading candidate to serve as the AG hormone of *M. rosenbergii*, we sought in this study to characterize *Mr-IAG* at the protein level in parallel to a study of the pattern of its gene expression throughout the animal life cycle. We, thus, produced a recombinant *Mr-IAG* and raised an antibody against it. The antibody specifically recognized a *∼*20 kDa protein in an AG extract and also reacted specifically with the AG cytoplasm. The presence of *Mr-IAG* was verified in a single gland by using mass spectrometry. The *Mr-IAG* transcript was expressed as early as 20 days after metamorphosis, prior to the emergence of external sex characters. Strikingly, the *Mr-IAG *relative transcript level was higher in the reproductively active morphotypes, the small males and blue-clawed males, than in the less reproductively active morphotype, the orange-clawed males, thus suggesting a key role for *Mr-IAG* not only in early sex differentiation but also in phenotypic differentiation and in the reproductive readiness of the different morphotypes.

## 2. Materials and Methods

### 2.1. Animals

*M. rosenbergii* were maintained at Ben-Gurion University of the Negev under the following conditions: Food comprising shrimp pellets (Rangen Inc., Buhl, ID, USA, 30% protein) was supplied *ad libitum* three times a week. Water quality was assured by circulating the entire volume through a biofilter maintaining all the required water physicochemical parameters, as described before [36]. Egg-berried females were separately transferred to closed 100-L tanks containing 12–16 ppt salt water, which was circulated through a 100-*μ*m mesh net. After hatching had occurred, the females were removed from the tanks, and the newly hatched larvae were maintained according to the protocol devised by Uno and Chin Soo [37] and fed with *Artemia *nauplii daily. Prawns were also sampled from two other populations, one grown by Ms. Ayana Perlberg at Dor Research Center and another by Dr. Shmuel Parnes of the ornamental aquaculture company Colors.

### 2.2. Tissue Preparation

#### 2.2.1. For Histology

AGs from mature male morphotypes were dissected out together with the attached terminal ampullae. Tissue samples were fixed in modified Carnoy's II for 48 h and dehydrated gradually through a series of increasing alcohol concentrations. Tissues were cleared and embedded in Paraplast (Kendall, MA, USA) according to conventional procedures. Sections of 5 *μ*m were cut onto silane-coated slides (Menzel-Gläser, Braunschweig, Germany). These slides served for all histological procedures, including immunohistochemistry and *in situ* hybridization. Prior to the histological procedures, the slides were deparaffinized in xylene and rehydrated gradually through a series of decreasing alcohol concentrations.

#### 2.2.2. For MS/MS

Single AGs of blue-clawed *M. rosenbergii* males were homogenized in 2 mM ammonium bicarbonate (AmBC) buffer, and protein content was determined using the method of Bradford [38]. Total proteins, 50 *μ*g, were denatured by 10 min of incubation at 70°C in 6 M urea and 10 mM dithiothreitol in 20 mM AmBC, followed by alkylation with 55 mM iodoacetamide at 25°C for 30 min. Thereafter, 1 *μ*g trypsin (Promega Corp., Madison, WI, USA) in 30 *μ*L were added to the sample, and overnight protein digestion was performed at 37°C.

### 2.3. Recombinant *Mr-IAG* Production

BL-21 bacteria were transformed with a pET-28A vector which includes a His-tag with a pro-*Mr-IAG* insert (the nucleotide sequence encoding for amino acids 28–173; accession #ACJ38227). The bacteria were grown in Luria-Bertani (LB) liquid medium and 50 *μ*g/mL kanamycin overnight at 37°C with shaking at 250 rpm. The bacteria were then diluted 1 : 100 into 1 L LB with kanamycin and grown under the same conditions to OD_600_ = 0.6. Thereafter, incubation proceeded overnight with 0.1 mM isopropyl-*β*-D-thiogalactopyranoside (IPTG) at 20°C. The cells were pelleted by centrifugation at 12,000 g for 5 min, and resuspended in 10 mL of lysis buffer containing 50 mM Tris-HCl (pH = 8), 2 mM EDTA and 1 mg lysozyme. The suspension was frozen (−80°C) and thawed (37°C) three times, followed by DNase treatment (0.2 mg with 20 mM MgCl_2_) for 30 min at 37°C. Inclusion bodies were then pelleted by centrifugation at 20,000 g for 30 min, and the protein pellet was solubilized with 4 mL of solubilization buffer (6 M guanidinium hydrochloride, 0.2 M NaCl, 0.1 M Tris-HCl pH = 8.3), supplemented with 0.01 M *β*-mercaptoethanol and incubated overnight at 4°C. The His-tagged protein was purified using Ni-NTA column according to manufacturer's protocol (QIAGEN, Hilden, Germany).

### 2.4. Polyclonal Antibody Production

A male New Zealand white rabbit was immunized with 250 *μ*g cleaned recombinant *Mr-IAG* (1 mL) emulsified with same volume of Freud's complete adjuvant (Sigma, St. Louis, Mo, USA). The injections were repeated four times, at two weeks intervals, but with the last three emulsified in Freud's incomplete adjuvant (Sigma). Two weeks after the last injection, the rabbit was killed, and the serum was withdrawn.

### 2.5. Western Blotting

Proteins extracted from the AGs (50 *μ*g) and from cleaned recombinant *Mr-IAG* (10 *μ*g) were subjected to 15% SDS-PAGE and then electrotransferred onto a nitrocellulose membrane. Western blot analysis was performed using the ECL method with overnight incubation at 4°C with anti-*Mr-IAG* antiserum (1 : 1,000) as the first antibody. The second antibody was goat antirabbit horseradish peroxidase (HRP) conjugate (1 : 15,000). Other than the mentioned exceptions, the procedure was carried out according to the manufacturer's instructions in an EZ-ECL detection kit (Biological Industries, Beit Haemek, Israel). The signal was detected using a LAS-4000 chemiluminescence detection system (Fujifilm, Tokyo, Japan).

### 2.6. Mass Spectrometry

Tryptic peptides were purified on a C-18 column and dissolved in 0.1% formic acid. Nanoliquid chromatography and mass-spectrometry analysis was performed as described previously [39] using a 75-*μ*m internal diameter fused silica column, packed with C18 (New Objective, Woburn, MA, USA) connected to an Eksigent nano-LC system (Eksigent, Dublin, CA, USA). Mass spectra where acquired using an LTQ-ORBITRAP XL (Thermo Fisher Scientific, San Jose, CA, USA). Full MS and MS/MS fragmentation was performed in the data-dependent mode, which allows switching between MS and MS/MS analysis. After full MS acquisition in the orbitrap, the multiply charged top 6 abundant masses where chosen for CID MS/MS fragmentation in the LTQ under the following conditions: the CID fragmentation was performed at 35% collision energy and 30 ms activation time. Protein identification and validation were performed using Sequest and Mascot algorithms operated under Proteome Discoverer 1.0 (Thermo Fisher Scientific) using an unpublished database containing the *Mr-IAG* sequence, which allowed 0.8 Da and 10 ppm tolerance.

### 2.7. Immunohistochemistry

Consecutive sections were incubated with anti-*Mr-IAG* antiserum. Slides were subjected to 0.5 M sodium citrate (pH = 6) for 30 min at 95°C for antigen retrieval then washed in phosphate-buffered saline (PBS) for 5 min, blocked with 3% bovine serum albumin (BSA) in PBS for 60 min, and finally incubated overnight at 4°C with anti-*Mr-IAG* antiserum (1 : 1,000) or with normal goat serum as the negative control. After three additional washes with PBS, the sections were incubated for 60 min with fluorescein isothiocyanate- (FITC-) conjugated goat antirabbit IgG. The nonspecific signal was washed twice with PBS for 5 min, followed by staining of the nuclei with diamidino phenylindole (DAPI; 1 : 1,000) in PBS and 50% glycerol. The slides were then viewed by fluorescence microscopy.

### 2.8. Genomic DNA Extraction

Genomic DNA was extracted from 2–10 mg 70% ethanol-fixed tissue using REDExtract-N-Amp Tissue PCR Kit (Sigma), according to the manufacturer's instructions.

### 2.9. PCR of Female Specific and Positive Control Sequences

PCR was performed with 100 ng genomic DNA, 1 *μ*M forward primer and 1 *μ*M reverse primer (primer sequences are available upon request), 12.5 *μ*L Ready Mix REDTaq (Sigma) and water to a final volume of 25 *μ*L. The PCR conditions were: 35 cycles of 30 s at 94°C, 30 s at 56°C, and 60 s at 72°C. PCR products were electrophoresed on a 2% agarose gel and visualized under UV light with ethidium bromide.

### 2.10. RT-PCR of *Mr-IAG* and *β*-Actin

Complementary DNA was prepared by a reverse transcriptase reaction containing 1 *μ*g of total RNA, extracted from the cephalothoraxes of juvenile prawns or zoeae or from the bases of the fifth walking legs of mature animals, and M-MLV reverse transcriptase H minus (Promega), according to the manufacturer's instructions. The cDNA was then amplified by PCR, as previously described by Manor et al. [23], and *Mr-IAG* tissue-specific expression was demonstrated by using forward (5′-GACAGCGTGAGGAGAAGTCC-3′, nt 627–646) and reverse (5′-TATAGGACAGGGACGG GATG-3′, nt 770–789)* Mr-IAG* specific primers. *M. rosenbergii β*-actin, accession #AF221096, served as a positive control using specific forward (5′-GAGACCTTCAACACCCCAGC-3′) and reverse (5′-TAGGTGGTCTCGTGAATGCC-3′) primers.

### 2.11. In Situ Hybridization

Consecutive sections were used for hybridization with sense and antisense probes and for morphological observations (staining with hematoxylin and eosin). Digoxygenin- (DIG-) labeled oligo-nucleotides for antisense and sense probes corresponding to nucleotides 29–1745 of *Mr-IAG *cDNA were synthesized using SP6 and T7 RNA polymerases, and the probes were hydrolyzed to reduce their lengths to *∼*200 bases, as described in the DIG Application Manual (Roche Applied Science, Swiss). Hybridization was carried out as described previously by Ventura et al. [24].

## 3. Results

**Figure 1:**
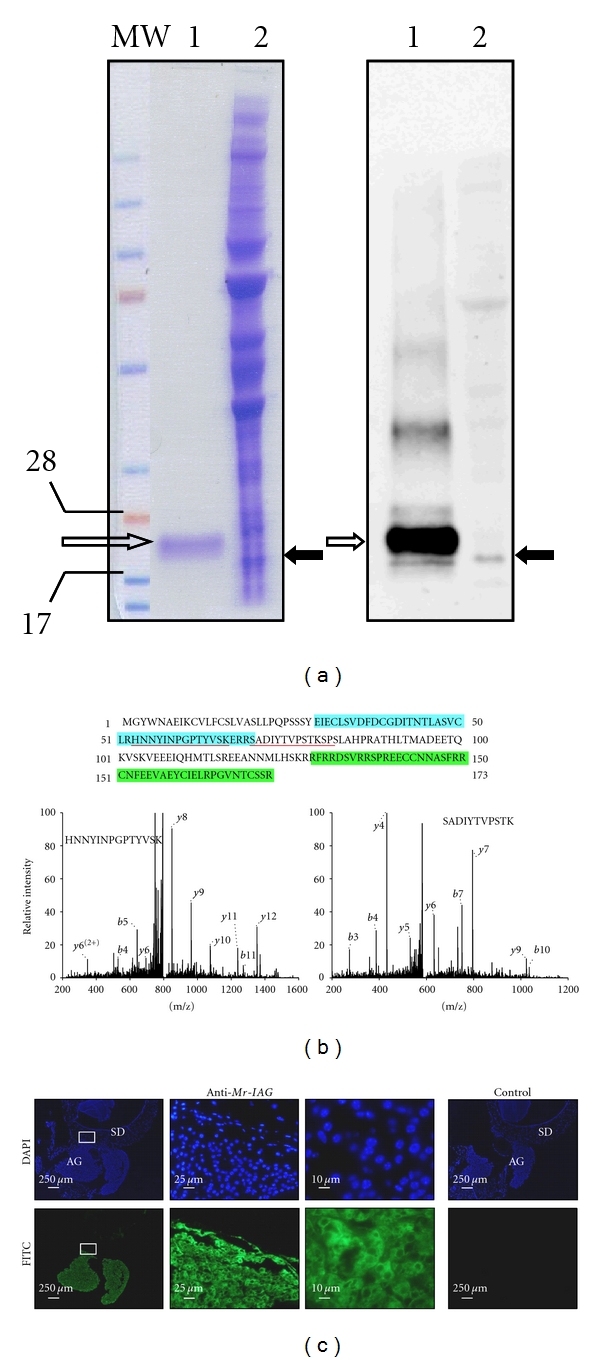
Identification of *Mr-IAG* in androgenic gland (AG) extracts and localization of the peptide in sections of the sperm duct (SD) and AG of adult *M. rosenbergii*. (a) Coommassie staining (left panel), and Western blot (right panel) of cleaned recombinant pro-*Mr-IAG* and AG extract (lanes 1 and 2, resp.). Black arrows indicate the recombinant pro-*Mr-IAG* (*∼*18.9 kDa). Open arrows indicate a band recognized by the antibody in the AG extract, corresponding to the estimated molecular weight of the endogenous propeptide (*∼*16.8 kDa). MW molecular weight size marker. (b) Identification of *Mr-IAG* by LC-MS. The identified peptides are underlined on the *Mr-IAG* sequence and the related peaks are indicated on the spectrum below. The *Mr-IAG* sequence shows the deduced signal peptide (italicized), followed the the Bchain (blue box), the C peptide, and the A chain (green box). Intensity maps are given for the *b* and *y* ions relevant to each peptide. (c) Immunohistochemical localization of *Mr-IAG*: from left to right are three increasing magnifications (×40, ×100, ×400) of SD and AG cross-sections incubated with anti-*Mr-IAG* antiserum. The SD and AG nuclei are stained blue with DAPI (upper panels). Specific signal (stained green with FITC) appears only in the cytoplasm of the AG cells (lower panels). To the right, no specific signal appears in the negative control sections, which were incubated with normal rabbit serum.

The presence of the protein encoded by *Mr-IAG* was demonstrated by using an antibody raised against recombinant pro-Mr-IAG in rabbit, which recognized a specific band of *∼*20 kDa both in the cleaned recombinant protein and in extracts from hypertrophied AGs (Figure 1(a); lanes 1 and 2, resp.). MS analysis of a single AG tryptic-digest revealed two peptides corresponding to amino acids (a.a.) 53–66 in the B-chain and a.a. 70–80 in the C peptide of *Mr-IAG* (Figure 1(b)). The scores calculated for these two peptides were 3.32 and 2.82, respectively. Localization of the protein was confirmed through immunohistochemical analysis of sperm ducts with attached hypertrophied AGs (cross sections) by using rabbit anti-*Mr-IAG* antiserum and a secondary goat antirabbit FITC-antibody. A strong signal restricted to the cytoplasm of the AG cells was obtained (Figure 1(c); AG), as can be clearly seen in the high magnitude sections (Figure 1(c); bottom, third from the left). No signal was detected in the sperm duct (Figure 1(c); SD) or in the other cells surrounding the AG; the nuclei of all these cells are stained blue with DAPI. The negative control comprised sections that were incubated with normal rabbit serum and not with anti-*Mr-IAG* antiserum. The AG-specific signal, taken together with the western blot hybridization result, confirmed the specific binding of our anti-*Mr-IAG* antiserum to the endogenous protein encoded by the *Mr-IAG* gene. 

**Figure 2:**
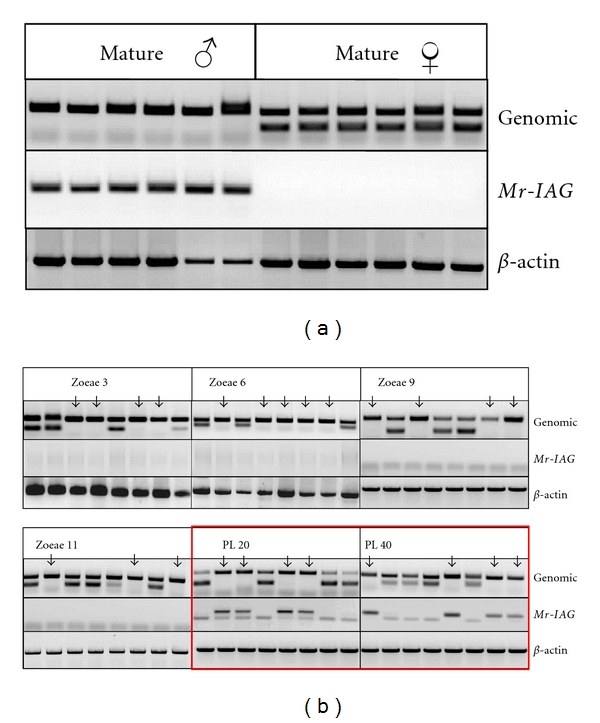
PCR of genomic DNA and RT-PCR of RNA extracted from larvae and juvenile and mature male and female* M. rosenbergii* individuals. Genomic-based PCR indicates maleness, where no female specific marker was amplified. RT-PCR was applied for detection of *Mr-IAG* expression by using *Mr-IAG* specific primers and **β*-actin* as positive control. (a) For adult male and female individuals, there is a correlation between the absence of the female specific marker and *Mr-IAG* expression. On the basis of this result, larvae and juveniles were defined as males according to the absence of the genomic sex marker (marked by arrows in Figure 2(b)). (b) None of the individuals sampled at larval stages (zoeae 3, 6, 9, and 11) expressed *Mr-IAG* regardless of the presence or absence of the genomic sex marker. At PL_20_ and onwards, all individuals identified as males according to absence of the female specific marker also expressed *Mr-IAG*.

For adult male (identified by external sex characters, such as male genital openings and appendix masculina) and female prawns there was a correlation between the absence of the female-specific sequence in male genomic extracts and *Mr-IAG* expression in RNA extracted from the bases of the fifth walking legs (Figure 2(a)). These findings confirmed that the female genomic marker can serve as a tool for the identification of males and females prior to the development of clear phenotypic sexual characters. When this tool was used for defining the temporal expression pattern of *Mr-IAG*, a correlation between individuals identified as males, according to the absence of the female-specific sequence, and the expression of the male specific transcript (*Mr-IAG*) was recorded in the early postlarval stages (Figure 2(b); PL_20_, and PL_40_). For the earlier larval stages, we did not find any individual expressing *Mr-IAG* (Figure 2(b); zoeae 3, 6, 9, and 11).

**Figure 3:**
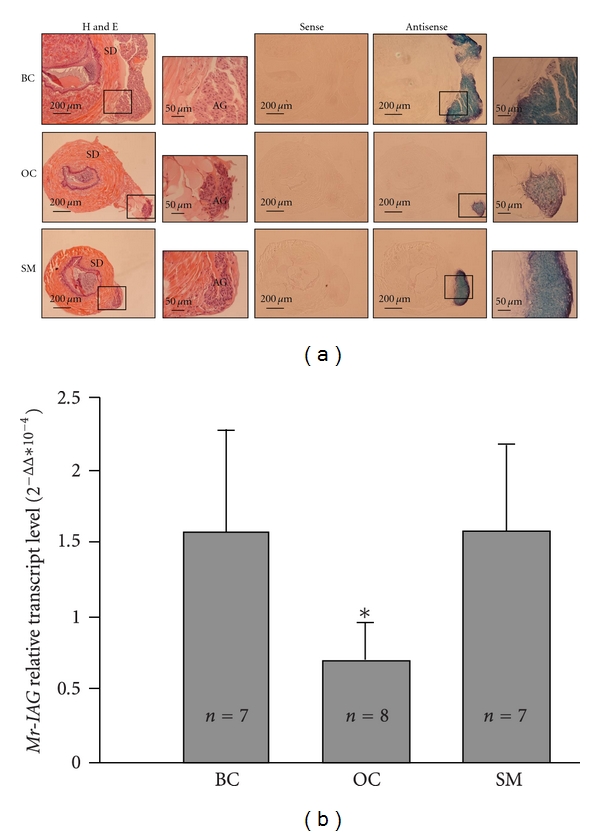
*In situ* hybridization and relative quantification of *Mr-IAG* in *M. rosenbergii* male morphotypes. (a) *In situ* hybridization in cross-sections of a sperm duct (SD) and attached androgenic gland (AG) in (top to bottom) blue-clawed male (BC), orange-clawed male (OC), and small male (SM). From left to right sections stained with hematoxylin and eosin (H&E) with the AG enlarged, the sense probe that served as a negative control, and the antisense *Mr-IAG* probe that hybridized specifically with the AG cells, also shown in higher magnification. (b) *Mr-IAG* relative expression level is significantly higher in blue-clawed males (BC) and small males (SM) than in the less reproductively active morphotype, the orange-clawed male (OC). Error bar represents standard deviation; asterisk represents significant difference (Mann-Whitney *U* test, *P* < .02).

In the adult phase, localization of *Mr-IAG* expression *in situ* confirmed AG-specific expression in all three male morphotypes (Figure 3(a)). A strong specific signal was detected exclusively in the AG cells by using an antisense probe. No signal was detected when the sense-strand probe was employed, further strengthening the notion that the specific hybridization of the antisense probe is to *Mr-IAG* RNA. Although the hybridization was clearly evident and specific in all three male morphotypes, the sections showed that the area occupied by the AG was relatively larger in the blue-clawed male and small male than in the orange-clawed male. Furthermore, the size of the AG relative to the sperm duct total diameter was much smaller in orange-clawed males than in blue-clawed males and in small males. Since these differences in the size of the AG suggested possible differences in *Mr-IAG* expression levels between the morphotypes, real-time PCR quantitative measurements were performed. Real-time RT-PCR revealed that *Mr-IAG* relative expression level was significantly higher in the blue-clawed and small males than in the less-reproductively active morphotype, the orange-clawed male (Figure 3(b); Mann-Whitney *U* test, *P* < .02).

## 4. Discussion

Over two decades ago, an active AG protein extract was purified in an isopod [40], leading to extensive research [41–47] culminating in the characterization of the peptide [21, 22] as an AG hormone [48] and in the identification of two orthologous genes in two other isopod species [49]. Since that time, despite extensive research efforts, no progress was made in revealing hormones in the important group of decapod crustaceans until the identification in the freshwater crayfish *Cherax quadricarinatus* of *Cq-IAG*, an AG specific transcript predicted to give rise to an insulin-like peptide, believed to be an AG hormone [23]. That study paved the way towards the identification of such a transcript in *M. rosenbergii* (*Mr-IAG*), thereby strengthening the premise that this peptide might be an AG hormone; this notion was also based on the demasculinizing effect of *Mr-IAG* silencing [24]. The present study gives the first glimpse into *Mr-IAG* at the protein level. The specific recognition by anti-*Mr-IAG* antiserum of a *∼*20-kDa peptide (Figure 1(a)) from an extract of AGs in *M. rosenbergii* is in keeping with the predicted pro-*Mr-IAG* (16.8 kDa). These molecular masses can be paralleled to earlier studies in Isopoda where the AG hormone precursor was identified and determined to be of similar size (16.5 kDa) [21]. MS analysis and identification of peptides corresponding to parts of the B-chain and C-peptide from the total protein digest of a single AG is given for the first time in this study (Figure 1(b)). The immunolocalization of *Mr-IAG* in the AG cytoplasm is in agreement with previous studies both in isopods [50] and decapods [51].

**Figure 4:**
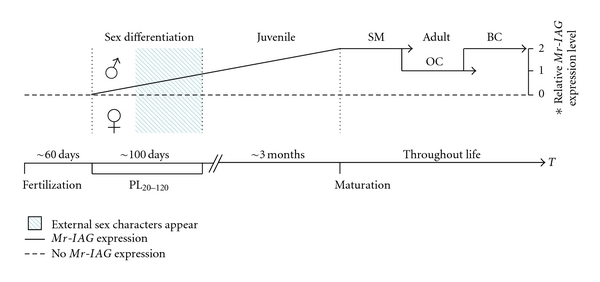
Schematic representation of the hypothetical *Mr-IAG* expression pattern during the lifespan of a *M. rosenbergii* individual. The time line (T) of an individual starts with fertilization, when there is no *Mr-IAG* expression (dashed line), goes through the earliest known time point of sexual differentiation, with males, but not females, exhibiting *Mr-IAG* expression (solid line, ♂). Immediately after the fork, the individuals continue to differentiate as manifested by the appearance of external sex characters at PL_20-120_; thereafter, the individuals go through a juvenile stage, which lasts for 3–5 months, then to adulthood when the males can be distinctly categorized into one out of three successive morphotypes: small males (SM), orange-clawed males (OC), and blue-clawed males (BC). Relative *Mr-IAG* expression level is twofold higher in the reproductively active morphotypes (SM and BC) than in the less reproductively active morphotype (OC). Asterisk denotes that the relative *Mr-IAG* expression level is confined to the adulthood period.

Until the present study, there has been no thorough characterization of the temporal expression pattern of any of the AG specific insulin-like peptides, whether predicted or proven [21–23, 49, 51]. The first predicted AG specific insulin-like peptide identified in Decapoda (*Cq-IAG*; [23]) was shown to retain its male specificity at early stages, but the lack of molecular tools prohibited the identification of males prior to the appearance of morphological secondary characters, and therefore it made it impossible to reveal an earlier time point at which *Cq-IAG* is expressed. This limitation is surmounted in this study with the use of a female-specific genomic sex marker (Figure 2). Use of this marker as a tool enabled the determination of the earliest time of expression of *Mr-IAG* at PL_20_ (Figure 2(b)), suggesting that PL_20_ is the stage at which there is a forking into male/female differentiation (Figure 4).

Since it was hypothesized that *Mr-IAG* is a key regulator of sex differentiation [24], its level should be tested for correlation with reproductive readiness during the adult phase. The morphotypic differentiation in *M. rosenbergii* adult males is a good model to test this correlation. The expression level of *Mr-IAG* transcript was found to be relatively higher in the dominant blue-clawed males and subordinate small males than in the reproductively less active orange-clawed males (Figure 3(b)). This result indicates that *Mr-IAG* is indeed a possible key regulator of morphotypic differentiation, which is known to rely upon the AG [34]. The expression level of *Mr-IAG* transcript in the different morphotypes is in keeping with the distinct reproductive behaviors of the small males, which practice a form of active sneak mating consistent with their small size and high mobility, and of the dominant blue-clawed males that actively court and protect the receptive females prior to mating [32]. We assume that the reduced rate of reproductive activity demonstrated by the orange-clawed males in the presence of dominant males is affected by an endocrine axis, as reflected in reduced AG hormone levels, similar to the situation in vertebrate species, where the reproductive behavior of male morphotypes is correlated with circulating androgen levels [27, 28]. The reduced levels of *Mr-IAG* in orange-clawed males is also in keeping with their reduced GSI compared with the reproductively active morphotypes [33]. Since *Mr-IAG* was previously shown to be crucial for male gonad activity [24], it is possible that that the *Mr-IAG* level is responsible for regulating the GSI.

Based on the accumulated data, Figure 4 presents a hypothetical scheme of the *M. rosenbergii* lifespan. The first part of diagram starts from fertilization and continues to around PL_20_. This point marks the earliest known time of male sexual differentiation in *M. rosenbergii*; it precedes the appearance of external sex characters, that is, the male gonopores and, the appendix masculina, which appear in *M. rosenbergii* in the time range of PL_20–120_. In the closely related penaeid shrimp *Litopenaeus vannamei* the appendix masculina emerges in males 50–90 days after metamorphosis, again with a certain size and weight threshold. The relatively late appearance of these characters in *Litopenaeus vannamei* compared with *M. rosenbergii* may be attributed to the fact that, contrary to *M. rosenbergii*, females grow faster than males in *Litopenaeus vannamei* [52]. The first anatomical evidence of sexual differentiation in *Litopenaeus vannamei*—the fully recognizable genital organ—becomes apparent at PL_16_ [53]. Returning to Figure 4, we hypothesize further that from PL_20_ onwards *Mr-IAG *is continuously expressed in males (Figure 4, solid line). It is in this time span, PL_20–120_, that the external sex characters appear. Sexual differentiation is followed by the differentiation of juvenile males or females, which takes three- to- five months in *M. rosenbergii* [54]. Adulthood in *M. rosenbergii* includes the successive morphotypic differentiation in males [31] that, according to the results of the present study, is characterized by a twofold increase in *Mr-IAG* expression in the reproductively active morphotypes versus the less reproductively active morphotype (small males and blue-clawed males versus orange-clawed males; Figure 4). 

To conclude, the present study provides circumstantial evidence indicating that *Mr-IAG* is a central AG hormone regulating both male sexual and morphotypic differentiation in *M. rosenbergii* and that the function of this hormone could parallel the dual role of androgens in vertebrate male sexual and morphotypic differentiation. Furthermore, this study sheds light on the mechanism underlying sex differentiation in invertebrates.

In practical terms, the results of this study reveal the temporal expression pattern of *Mr-IAG* and further strengthen the belief that it is a major AG hormone during early *M. rosenbergii* sexual differentiation. Since *M. rosenbergii* is a commercially important species, with a clear advantage in growing all-male populations [25, 55], the results of the present study might prove useful in the establishment of future biotechnologies for better management of cultured all-male populations.
